# Effects of lifestyle education program for type 2 diabetes patients in clinics: a cluster randomized controlled trial

**DOI:** 10.1186/1471-2458-13-467

**Published:** 2013-05-14

**Authors:** Misa Adachi, Kazue Yamaoka, Mariko Watanabe, Masako Nishikawa, Itsuro Kobayashi, Eisuke Hida, Toshiro Tango

**Affiliations:** 1 Nutrition Support Network LLC, 2-2-4 Wakamatu, Sagamihara, Kanagawa, 252-0334, Japan; 2 Teikyo University, Graduate School of Public Health, 2-11-1 Kaga, Itabashi-ku, Tokyo, 173-8605, Japan; 3 Showa Women’s University, Graduate School of Life Science, 1-7 Taishido, Setagaya, Tokyo, 154-0004, Japan; 4 Department of Technology Assessment and Biostatistics, National Institute of Public Health, 2-3-6 Minami, Wako, Saitama, 351-0197, Japan; 5 Kobayashi Medical Clinic, Ryokujukai Cooperation, 5-27-28 Sagamiono, Sagamihara, Kanagawa, 252-0303, Japan; 6 Center for Medical Statistics, SAN Building 401, 2-9-6 Higashi Shinbashi, Minato-ku, Tokyo, 105-0021, Japan

**Keywords:** Diabetes type 2, Lifestyle education, Cluster randomized trial, Dietary habit, Food frequency questionnaire

## Abstract

**Background:**

The prevalence of type 2 diabetes is rising worldwide, as has been the global mean fasting plasma glucose level. This study aimed to evaluate the effectiveness of a structured individual-based lifestyle education (SILE) program to reduce the hemoglobin A1c (HbA_1c_) level in type 2 diabetes patients delivered by registered dietitians in primary care clinical settings.

**Methods:**

This was a 6-month prospective cluster randomized controlled trial in a primary care setting with randomization at the practice level. Twenty general practitioners in 20 clinics in Kanagawa prefecture, Japan, were involved. 193 adults (51% men, mean age 61.3 years) with type 2 diabetes and HbA_1c_ ≥6.5% who received treatment in medical clinics were the participants. A SILE program was implemented through 4 sessions with trained registered dietitians during the 6-month study period. Results were compared with those of a control group who received usual care. The primary endpoint was the change in HbA_1c_ levels at 6 months from baseline. Secondary endpoints were the changes at 6 months from baseline in fasting plasma glucose, lipid profile, blood pressure, BMI, energy, and nutrient intakes (whole day and each meal). Intention-to-treat analysis was conducted. Mixed-effects linear models were used to examine the effects of the treatment.

**Results:**

The mean change at 6 months from baseline in HbA_1c_ was a 0.7% decrease in the intervention group (n = 100) and a 0.2% decrease in the control group (n = 93) (difference −0.5%, 95%CI: -0.2% to −0.8%, p = 0.004). After adjusting for baseline values and other factors, the difference was still significant (p = 0.003 ~ 0.011). The intervention group had a significantly greater decrease in mean energy intake at dinner compared with the control group and a greater increase in mean vegetable intake for the whole day, breakfast, and lunch as shown in crude and adjusted models. A tendency toward improvement was observed in the other secondary endpoints but the improvement was not statistically significant. These results were confirmed by several sensitivity analyses.

**Conclusions:**

The SILE program that was provided in primary care settings for patients with type 2 diabetes resulted in greater improvement in HbA_1c_ levels than usual diabetes care and education.

**Trial registration:**

http://UMIN000004049

## Background

The prevalence of type 2 diabetes is rising worldwide, as has been the global mean fasting plasma glucose (FPG) level [1]. Type 2 diabetes is associated with serious complications such as blindness and renal failure, as well as an increased risk of cardiovascular disease [2]. Type 2 diabetes is responsible for a disproportionate use of health service resources, and its increased prevalence presents a serious problem from the viewpoint of medical economics. In Japan, approximately 8.9 million people are estimated to have diabetes based on a hemoglobin A1c (HbA_1c_) level of 6.5% (NGSP) or over or receiving treatment for type 2 diabetes [3]. Lifestyle modification is the cornerstone of treatment for people with type 2 diabetes. Little evidence of benefit exists for nutritional education in patients with type 2 diabetes in clinics in community medicine settings. Lack of a useful tool to assess dietary intake and difficulty in continuous management of a patient’s lifestyle may be related to this.

Meta-analyses of randomized controlled trials (RCTs) have shown that lifestyle interventions improved glycemic control with type 2 diabetes patients [4]. Although several types of lifestyle education programs have been proposed, reports of their effectiveness in Asian populations, including Japanese populations, have been scant [5,6]. Considering that a frequent feature of type 2 diabetes in Japan is not obesity, dietary education that focuses on the pattern of eating to improve the HbA_1c_ level by controlling the postprandial rise in plasma glucose and by improving the fasting plasma glucose level is important. Namely, modifying energy intake at dinner and increasing vegetable intake at breakfast and lunch should be effective. The rationale for this strategy is based on the following: 1) proper energy intake at dinner is important since night-time activity is less than day-time activity considering circadian rhythms [7]; 2) a meal with increased dietary fiber can control the postprandial rise in the plasma glucose level and contribute to the improvement in HbA_1c_[8]; and 3) to increase vegetable intake at breakfast and lunch is required because vegetable intake at these meals is usually less than at dinner. Therefore, to conduct effective dietary education, an appropriate assessment of nutritional intake at each meal is important.

A food frequency questionnaire (FFQ) is a feasible method for this purpose, and we developed a FFQ consisting of a list of 82 foods (FFQW82) [9]. Using the FFQW82, we have recently developed a structured individual-based lifestyle education (SILE) program to be used in clinics that aims to modify dietary intake at breakfast, lunch, and dinner and that is focused on behavior assessment, goal-setting, problem-solving, and provision of tailored information from registered dietitians. The main current recommendations for individuals with diabetes by the Japan Diabetes Society (JDS) [10] related to energy intake are calculated using the ideal body weight and three levels of physical activity. The proportion of total energy intake from carbohydrate is recommended to be 50 ~ 60% and from total fat less than 25%, from saturated fatty acid less than 7%, and from polyunsaturated fatty acid less than 10%. Intakes of protein, fiber, and salt are recommended to be 1.0 ~ 1.2 g/kg/day, 20 ~ 25 g/day, and less than 10 g/day, respectively. Our SILE program basically follows the guidelines of the JDS with the additional recommendation to reduce energy intake at dinner and increase vegetable intake at breakfast and lunch, which should be a more practical strategy than the JDS recommendations.

The aim of this study was to examine the effect of lifestyle education using the SILE program provided by registered dietitians for type 2 diabetes patients in primary care clinics by assessing changes in HbA_1c_ levels, other clinical data and dietary intakes. Results will provide useful information for not only Japanese type 2 diabetes patients but also for type 2 diabetes patients in other Asian countries and for patients in Western countries who have already modified their dietary intake to some extent, although still insufficiently, or who have failed with regard to weight control in many instances.

## Methods

### Study design

This was a 6-month cluster randomized controlled trial with two intervention arms performed between September 2007 and June 2011 inclusive at clinics in Kanagawa prefecture, Japan. Details of the study design and calculation of the sample size were published previously [11], with only a brief description shown below. The study was approved by the Medical Ethical Committee of the National Institute of Public Health in Japan in 2006 (NO. NIPH-IBRA #06005).

### Participants

Volunteer general practitioners who agreed with our study purposes and procedures were recruited. We randomly assigned in turn a general practitioner representing a primary care clinic to either the intervention group (IG) or control group (CG) with the use of a randomization list (random permutated blocks with block size 2). Participating general practitioners in each primary care clinic examined the study patients themselves. 20 clinics were finally randomized to either the IG or CG. Participating general practitioners were encouraged to continuously recruit all eligible study patients from September 2007 to the end of December 2010. Each general practitioner was asked to recruit no fewer than 10 patients, if possible, but no more than 13 who satisfied the criteria for eligibility to participate in the study. Our study protocol called for 10 patients per clinic; however in practice we permitted a range of 7–13 patients per clinic. The higher number was allowed to maintain statistical power in the event of dropouts and the lower number was permitted in clinics where in 10 patients could not be enrolled.

Study participants were men and women from 20 to 79 years of age with type 2 diabetes and HbA_1c_ concentrations of 6.5% (NGSP) or over and who were receiving treatment by the assigned general practitioner in the primary care clinic. The value for HbA_1c_ (%) was estimated as a National Glycohemoglobin Standardization Program (NGSP) equivalent value (%) calculated by the formula HbA_1c_ (%) = HbA_1c_ Japan Diabetes Society (JDS) (%) + 0.4%, considering the relational expression of HbA_1c_ (JDS) (%) measured by the previous Japanese standard substance and measurement methods and HbA_1c_ (NGSP) [12]. Therefore, 6.5% (NGSP in the protocol paper [11]) is equivalent to 6.1% (JDS in this paper).

### Intervention and control groups

The IG received structured individual-based lifestyle education that mainly encouraged the reduction in energy intake at dinner and an increase in vegetable intake at breakfast and lunch. Support for self-management of glycemic control, such as by diet, exercise, and stress management, was provided in 3 or 4 sessions with trained registered dietitians during the study period. The program for the IG was structured in four steps: “Basic information on glycemic control”, “Actions for glycemic control”, “Daily activities for glycemic control”, and “Management of stress for glycemic control”. We developed this program based on some of the strategies described in previous studies [13-15]. An assessment sheet, which was developed by consulting evidence-based practice guidelines for treatment of diabetes in Japan [15], was used. Patients decided on one or two short-term goals for glycemic control to be achieved in the next month based on the results of the FFQW82 and advice by registered dietitians. Sedentary participants were encouraged to increase basal physical activity. We recommended a gradual increase in physical activity in daily life rather than a formal fitness regimen or sports activity during leisure time. Before the start of the study, registered dietitians had the opportunity to gain experience with the intervention protocol under supervision by the project team.

Patients in the CG received information on dietary intake estimated using the FFQW82 and general advice on glycemic control by registered dietitians. A consultation by a dietitian was provided once with a month of randomization. The general advice was that which was usually given for glycemic control usually given by a general practitioner or a nurse in a clinic, such as “don’t eat too much eat at dinner”, “eat more vegetables”, “do exercise”, and so on. As for clinics without dietitians (11 clinics; 6 for IG, 5 for CG), registered dietitians were randomly allocated. Four registered dietitians visited more than one of these clinics. Training of registered dietitians was conducted based on an instruction manual and thus did not differ between those at the intervention clinics and control clinics.

### Study hypothesis

The hypothesis underlying the study is that participation in the IG would decrease the HbA_1c_ level by 15% from baseline (primary endpoint) after 6 months whereas we assumed that such a decrease would not occur in the CG. The value of a 15% decrease in the HbA_1c_ level was decided through reference to materials published in the USA [13] and through our experience with a previous survey [16].

### Outcome measures

The primary endpoint was a change from the baseline HbA_1c_ level after 6 months of education. Secondary endpoints were changes at 6 months from baseline in other clinical data such as body mass index (BMI), blood pressure, fasting plasma glucose (FPG), and the lipid profiles (low density lipoprotein [LDL], high density lipoprotein [HDL], triglycerides [TG]). Dietary intakes were assessed using the FFQW82. Energy and vegetable intakes (whole day and each meal) and dietry fiber intake (whole day), proportions of carbohydrate, protein, and fat to total energy intakes were examined as secondary endpoints.

Measurements were made at baseline, at 3 months, and at 6 months (endpoint). However, because the changes in the endpoints between the IG and CG were the effect sizes in the present study, we used the measures at 3 months only for the last observation carried forward **(**LOCF) analysis.

All clinical data were obtained by general practitioners in the course of their usual clinical practice and by their generally used methods. We then extracted these data from their medical records. Our protocol stated that waist circumference would be a secondary outcome measure, but waist circumference measurements were missing for some patients, and there appeared to be large variations in the measurements for some individuals. Thus we eliminated waist circumference from the analysis. We assessed physical activity levels at baseline and at 6 months using the question “How frequently do you engage in physical activity or aerobic exercise such as walking, swimming, physical training, etc. for 10 minutes or more in a week?” The response was classified into 3 levels: “none”, “1-3 day(s) per week”, and “4 days or more per week”. Because the SILE program concentrated on dietary modifications, we used the levels of physical activity at baseline as an adjusted variable in the multivariate adjusted model (Model 4). Changes in levels of physical activity could affect outcomes. We added such changes in the multivariate adjusted model (Model 4).

### Statistical analysis

Pearson correlation coefficients for changes in outcome measures were obtained.

Intention-to-treat (ITT) analysis was conducted. The LOCF method and a multiple imputation (MI) method using chained equations under the assumption of missing at random (MAR) [17] were used for handling missing data. Per-protocol analysis (PPA) with the complete data set (CDS) was conducted as a sensitivity analysis. Mixed-effects linear models were used to examine the effects of the treatment and cluster effect. Namely, in order to assess within-clinic (S^2^_w_) and between-clinic (S^2^_b_) variances, mixed-effects linear models were used, which included a crude model (Model 1), a baseline-adjusted model (Model 2), baseline, gender, age, and BMI-adjusted model (Model 3), and multivariate-adjusted (gender, age, BMI, smoking habit, baseline and change in physical activity level, family history of type 2 diabetes, and complications) model (Model 4) using individual data.

A significance level of 5% (two sided) was used for all tests. All statistical analyses were performed using SAS version 9.2 for Windows (SAS Institute, Inc., Cary, NC, USA).

## Results

**Figure 1:**
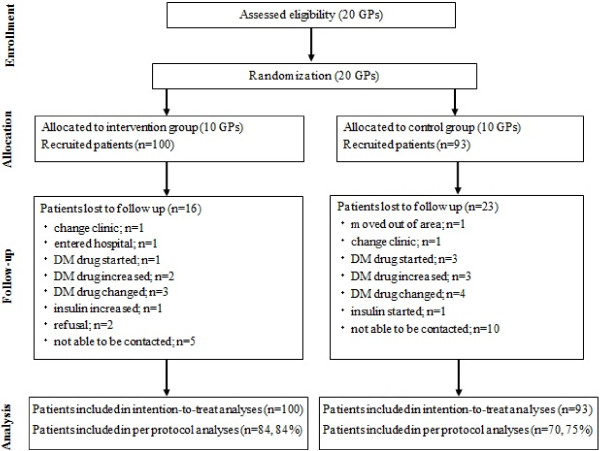
Flow diagram of progress of clusters and individuals through phases of the randomized trial.

Following the guidelines of CONSORT [18], Figure 1 shows a flow diagram of progress of the clusters and individuals through phases of the randomized trial. In total, 20 clinics were randomized to either the IG (10 clinics) or CG (10 clinics), and 215 patients (113 IG, 102 CG) were recruited. Eleven patients (5 IG, 6 CG) did not meet the inclusion criteria and 11 patients (8 IG, 3 CG) could not complete the FFQW82. Finally, 193 patients were deemed eligible and assigned to their respective groups (100 IG; 93 CG) according to the allocation of the clinic where they received treatment. Therefore 193 patients were used for ITT analysis. During the study, 39 patients discontinued participation because they had left the area, changed clinics, became hospitalized, increased dosage or changed hypoglycemic agents, started or changed dosage of insulin, refused to continue, or could no longer be contacted (see Figure 1). Thus, PPA included 154 patients (84 [80%] IG, 70 [75%] CG). The background characteristics of participants in the IG and CG are shown in Table 1. Overall mean age was 61.3 years (60.4 years IG, 62.3 years CG). Table 2 shows baseline and 6-month follow-up statistics on the outcome measures in both study groups.

**Table 1:** Background characteristics of patients with type 2 diabetes allocated to intervention group or control group

	**Intervention group (n = 100)**	**Control group (n = 93)**
Age (yr)	60.4 (11.4)	62.3 (10.1)
Women	55 (55%)	54 (58%)
Smoking status		
Not smoking	88 (88%)	78 (84%)
Current smoking	12 (12%)	13 (14%)
Past smoking	0 (0%)	2 (2%)
Family history of type 2 diabetes		
Having	49 (49%)	34 (37%)
Not having	46 (46%)	48 (52%)
Unknown	5 (5%)	11 (12%)
Complications^$^		
None	14 (14%)	12 (12%)
Hypertension	59 (59%)	56 (60%)
Dyslipidemia	21 (21%)	22 (24%)
Diabetes treatment		
Diet only	36 (36%)	31 (33%)
Oral hypoglycemic only	54 (54%)	50 (54%)
Insulin and oral hypoglycemic	5 (5%)	5 (5%)
Insulin only	5 (5%)	7 (8%)
Other Medication^$^		
None	25 (25%)	39 (42%)
Antihypertensive	60 (60%)	39 (42%)
Lipid modifying	46 (46%)	33 (35%)

Data are mean (SD) or number (%).

$ multiple responses.

**Table 2:** Baseline and at 6th months clinical and dietary characteristics

	**Intervention group (n = 100)**						**Control group (n = 93)**					
	**Baseline**			**at 6th months**			**Baseline**			**at 6th months**		
**Characteristics**	**n**	**Mean**	SD*	**n**	**Mean**	SD*	**n**	**Mean**	SD*	**n**	**Mean**	SD*
HbA_1c_ (%)^#^	100	7.6	1.4	84	6.7	1.2	93	7.3	1.1	70	7.0	1.0
BMI (kg/m^2^)	100	26.3	4.6	91	25.6	4.3	93	24.9	4.6	82	24.5	4.4
Fasting plasma glucose (mg/dl)	79	174	70	78	145	63	77	160	71	66	134	48
Systolic blood pressure (mmHg)	100	133	17	85	132	15	93	132	17	81	130	16
Diastolic blood pressure (mmHg)	100	78	12	84	77	11	93	75	12	81	72	11
LDL cholesterol (mg/dl)	96	127	29	87	121	29	89	122	30	71	122	28
HDL cholesterol l (mg/dl)	87	56	16	80	58	20	88	56	13	68	59	15
Triglycerides (mg/dl)	84	151	84	78	135	104	77	141	74	69	133	70
Energy intakes												
Whole day (kcal)	100	1686	272	80	1624	220	93	1671	238	68	1655	259
Breakfast (kcal)	100	412	161	80	423	137	93	403	151	68	401	149
Lunch (kcal)	100	551	159	80	535	130	93	551	125	68	540	143
Dinner (kcal)	100	741	100	80	700	91	93	732	95	68	726	99
Proportion of carbohydrate (%)	100	55.3	2.5	80	55.2	2.4	93	55.1	2.9	68	54.6	4.2
Proportion of protein (%)	100	15.9	1.3	80	16.1	0.8	93	16.0	1.0	68	16.3	1.5
Proportion of fat (%)	100	28.8	2.0	80	28.7	2.0	93	28.9	2.3	68	29.2	3.1
Fiber intake (g)	100	12.1	2.3	80	12.8	2.1	93	12.6	2.1	68	12.7	2.5
Vegetable intakes												
Whole day (g)	100	196.5	[155, 242]	80	236.5	[202.0, 281.5]	93	223.0	[183, 275]	68	208.5	[175, 263.5]
Breakfast (g)	100	26.5	[8.0, 45.5]	80	42.0	[30.0, 65.5]	93	38.0	[13, 60]	68	40.0	[15.5, 52.5]
Lunch (g)	100	38.5	[20.5, 60]	80	58.0	[37.5, 78]	93	55.0	[28, 75]	68	44.0	[23, 69.5]
Dinner (g)	100	130.0	[118, 143.5]	80	137.0	[124.5, 148.5]	93	132.0	[120, 147]	68	134.0	[121, 147]
Exercise status	100			82			93			64		
None		54	54%		24	29%		35	38%		27	42%
1-3 day(s)/ wk		19	19%		14	17%		16	17%		7	11%
4 days or over/ wk		27	27%		44	54%		42	45%		30	47%
Exercise status: change from baseline	82						62					
Less					9	11%					13	20%
Not change					43	52%					40	63%
More					30	37%					11	17%

Dada are mean (SD), Median [25%tile, 75%tile], or number (%).

* SD: standard deviation.

^#^ Value of HbA_1C_ is JDS^15^.

The 6-month ITT/LOCF analysis showed a significantly greater mean change in HbA_1c_ from baseline (95% confidence interval) in the IG compared with the CG: -0.7% vs. -0.2%; difference −0.5% (−0.8% to −0.2%), p = 0.004 (Model 1). Those values for the baseline adjusted analysis were −0.6% vs. -0.2%; difference −0.5% (−0.8% to −0.2%), p = 0.004 (Model 2). Significant improvements were also shown for gender, age, and BMI adjusted analysis (Model 3) (p = 0.003) and for multivariate-adjusted analysis (Model 4) (p = 0.011). Further analyses of the HbA_1c_ level with PPA/CDS and MI method showed similar significant effects. A tendency toward improvement was observed but the improvement was not statistically significant for the other clinical outcome measures, such as BMI, FPG, systolic blood pressure, diastolic blood pressure, LDL, HDL, and TG (Table 3.) Because FPG, HDL, and TG included many missing data and showed a large variance, the MI solutions were not converged and the estimates by the MI method for those were not obtained.

**Table 3:** Mean change at 6th months from baseline in clinical data

	**Intervention**			**Control**			**Model 1 (crude)**			**Model 2 (adjusted)**			**Model 3 (adjusted)**			**Model 4 (adjusted)**		
	**Mean**	**±**	**SE**	**Mean**	**±**	**SE**	**Difference**	**95%CI**	***p*-value**	**Difference**	**95%CI**	***p*-value**	**Differences**	**95%CI**	***p*-value**	**Difference**	**95%CI**	***p*-value**
HbA_1c_(%)^#^																		
LOCF^1)^	−0.7	±	0.1	−0.2	±	0.1	−0.5	(−0.8 to −0.2)	0.004	−0.5	(−0.8 to −0.2)	0.004	−0.5	(−0.8 to −0.2)	0.003	−0.5	(−0.9 to −0.1)	0.011
CDS^2)^	−0.7	±	0.1	−0.2	±	0.1	−0.5	(−0.9 to −0.1)	0.009	−0.4	(−0.8 to −0.1)	0.014	−0.5	(−0.1 to −0.8)	0.013	−0.5	(−1.0 to −0.1)	0.028
MI^3)^	−0.7	±	0.1	−0.3	±	0.1	−0.5	(−0.9 to −0.1)	0.030	−0.4	(−0.8 to −0.1)	0.041	−0.4	(−0.8 to −0.2)	0.045	−0.4	(−0.6to −0.0)	0.045
BMI(kg/m^2^)																		
LOCF^1)^	−0.5	±	0.1	−0.3	±	0.2	−0.2	(−0.7 to 0.2)	0.351	−0.1	(−0.6 to 0.4)	0.598	−0.1	(−0.6 to 0.3)	0.548	−0.3	(−0.8 to 0.2)	0.221
CDS^2)^	−0.6	±	0.2	−0.3	±	0.2	−0.3	(−0.8 to 0.1)	0.146	−0.2	(−0.8 to 0.3)	0.331	−0.3	(−0.8 to 0.3)	0.297	−0.3	(−0.9 to 0.2)	0.216
MI^3)^	−0.5	±	0.1	−0.3	±	0.2	−0.2	(−0.7 to 0.2)	0.351	−0.1	(−0.7 to 0.5)	0.693	−0.1	(−0.7 to 0.4)	0.633	−0.1	(−0.6 to 0.5)	0.829
FPG(mg/dl)																		
LOCF^1)^	−19	±	8	−20	±	8	1	(−23 to 25)	0.919	8	(−11 to 28)	0.367	9	(−11 to 29)	0.347	22	(−10 to 54)	0.165
CDS^2)^	−20	±10	10	−14	±	11	7	(−37 to 23)	0.633	1	(−23 to 25)	0.933	0	(−25 to 25)	0.993	5	(−33 to 43)	0.792
MI^3) $^																		
SBP(mmHg)																		
LOCF^1)^	−1	±	3	0	±	3	1	(−9 to 7)	0.748	−1	(−7 to 6)	0.802	0	(−7 to 6)	0.963	0	(−8 to 8)	0.951
CDS^2)^	−1	±	3	−2	±	3	1	(−7 to 9)	0.804	2	(−5 to 9)	0.514	4	(−4 to 9)	0.416	4	(−5 to 12)	0.370
MI^3)^	−1	±	3	−2	±	3	−1	(−9 to 7)	0.738	−2	(−8 to 5)	0.593	−2	(−9 to 5)	0.523	−3	(−11 to 4)	0.368
DBP(mmHg)																		
LOCF^1)^	−1	±	1	−3	±	2	1	(−3 to 6)	0.560	−3	(−1 to 7)	0.164	−2	(−2 to 6)	0.239	4	(−1 to 8)	0.088
CDS^2)^	−0	±	1	−4	±	1	3	(−1 to 7)	0.159	4	(−0 to 8)	0.050	4	(−0 to 8)	0.073	5	(0 to 10)	0.049
MI^3)^	−1	±	1	−3	±	2	−2	(−7 to 2)	0.296	−3	(−8 to 1)	0.120	−3	(−8 to 1)	0.166	−3	(−8 to 2)	0.171
LDL(mg/dl)																		
LOCF^1)^	−5	±	2	−1	±	2	−4	(−10 to 3)	0.286	−2	( −8 to 3)	0.387	−3	(−10 to 3)	0.245	−4	(−13 to 5)	0.344
CDS^2)^	−4	±	2	0	±	3	−4	(−12 to 3)	0.241	−3	(−10 to 4)	0.371	−4	(−11 to 3)	0.252	−5	(−16 to 5)	0.283
MI^3)^	−5	±	4	2	±	4	7	( 2 to 12)	0.198	6	( 0 to 10)	0.250	7	(−4 to 17)	0.196	7	(−6 to 18)	0.223
HDL(mg/dl)																		
LOCF^1)^	−1	±	1	1	±	1	−0	(−4 to 3)	0.823	−0	(−4 to 3)	0.863	0	(−3 to 4)	0.861	2	(−3 to 8)	0.393
CDS^2)^	1	±	2	2	±	2	−1	(−6 to 4)	0.745	−1	(−6 to 4)	0.701	0	(−5 to 5)	0.994	2	(−3 to 8)	0.393
MI^3) $^																		
Triglycerides(mg/dl)																		
LOCF^1)^	−3	±	8	−5	±	9	2	(−23 to 26)	0.894	4	(−20 to 28)	0.748	2	(−22 to 27)	0.239	7	(−28 to 43)	0.667
CDS^2)^	−7	±	8	−5	±	9	−3	(−29 to 24)	0.833	4	(−27 to 25)	0.951	−5	(−31 to 22)	0.708	−1	(−38 to 36)	0.953
MI^3) $^																		

SE: standard error, 95%CI: 95% confidence interval, degree of freedom = 18.

^#^ Value of HbA_1C_ is JDS^15^.

1) LOCF: last observation carried forward (IG: n = 100, CG: n = 93).

2) CDS: complete data set. (IG: n = 84), CG: n = 70).

3) MI: Multiple imputation with all analysed variables (number of imputations = 200) (IG: n = 100, CG: n = 93).

Model 1: crude.

Model 2: mixed model adjusted for baseline.

Model 3: mixed model adjusted for baseline, gender, age and BMI.

Model 4: mixed model adjusted for baseline, gender, age, BMI, smoking status, exercise status, change of exercise level, family history of type 2 diabetes, and complication.

^$^ The MI models did not converge.

Larger energy intake at dinner (r = 0.29, p = 0.001) and larger fat intake at dinner (r = 0.17, p = 0.055) were correlated with an increase in the HbA_1c_ level whereas larger vegetable intake at breakfast (r = −0.21, p = 0.015) and for the whole day (r = −0.18, p = 0.042) were correlated with reductions in the HbA_1c_ level. Increases in BMI (r = 0.28, p < 0.001), FPG (0.47, p < 0.001), LDL (r = 0.22, p = 0.011), and TG (r = 0.34, p < 0.001) were correlated with increases in HbA_1c_. This suggests that the changes between endpoints were moderately correlated with each other.

**Figure 2:**
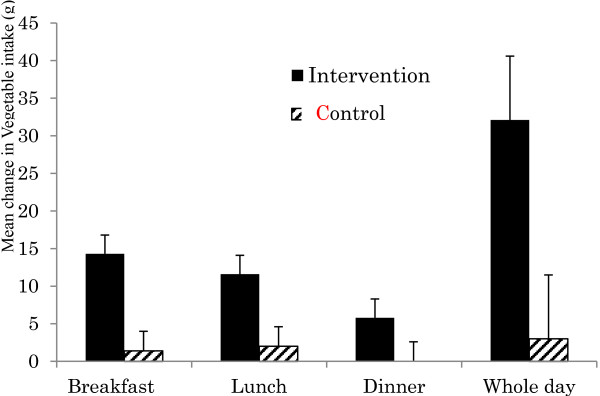
Mean change with standard error at 6 months from baseline in vegetable intake (g).

Results of the analyses for dietary outcomes are summarized in Table 4. There was a statistically significant difference at 6 months from baseline in energy intake at dinner in the IG compared with the CG when all models were used for both the ITT/LOCF and PPA/CDS analyses, but not MI method (Table 4). There was a statistically significant mean change at 6 months from baseline between groups in vegetable intake for a whole day, breakfast, and lunch (p = 0.000 ~ 0.043 for Model 1 to Model 3) (Figure 2). Specifically, intake of vegetables in the IG was significantly greater than in the CG for a whole day breakfast and lunch. For dietary fiber intake, a statistically significant increase was shown only for Model 1 and Model 2 by ITT/LOCF. No statistically significant changes were shown for the other nutrient outcomes such as proportions of carbohydrate, protein, and total fat of total energy.

**Table 4:** Mean change at 6th months from baseline in dietary data

	**Intervention**			**Control**			**Model 1 (crude)**			**Model 2 (adjusted)**			**Model 3 (adjusted)**			**Model 4 (adjusted)**		
	**Mean**	**±**	**SE**	**Mean**	**±**	**SE**	**Difference**	**95%CI**	***p*-value**	**Difference**	**95%CI**	***p*-value**	**Difference**	**95%CI**	***p*-value**	**Difference**	**95%CI**	***p*-value**
**Energy intake-whole day (kcal)**																		
LOCF^1)^	−29	±	16	−7	±	17	−22	(−23 to 67)	0.364	−21	(−19 to 61)	0.333	−23	(−18 to 64)	0.283	−27	(−112 to 9)	0.222
CDS^2)^	−54	±	22	9	±	24	−54	(−10 to 117)	0.114	−50	(− 1 to 108)	0.085	−54	(− 1 to 108)	0.073	−53	(−151 to −12)	0.034
MI^3)^	−54	±	35	−20	±	38	−34	(−76 to 145)	0.519	−33	(−68 to 134)	0.493	−39	(−62 to 140)	0.420	−52	(−159 to 50)	0.321
**Energy intake-breakfast (kcal)**																		
LOCF^1)^	19	±	11	10	±	12	8	(−41 to 24)	0.619	11	(−41 to 19)	0.480	11	(−41 to 20)	0.505	12	(−56 to 32)	0.598
CDS^2)^	17	±	15	23	±	17	6	(−39 to 51)	0.793	2	(−43 to 38)	0.909	0	(−42 to 41)	0.986	2	(−50 to 55)	0.922
MI^3)^	1	±	22	5	±	24	−5	(−59 to 68)	0.891	0	(−65 to 64)	0.988	−3	(−61 to 66)	0.937	2	(−72 to 62)	0.888
**Energy intake-lunch (kcal)**																		
LOCF^1)^	−6	±	10	−6	±	10	0	(−28 to 29)	0.974	−1	(−26 to 27)	0.965	−3	(−24 to 30)	0.829	22	(−17 to 62)	0.567
CDS^2)^	−13	±	13	−2	±	14	−11	(−27 to 49)	0.570	−11	(−27 to 49)	0.551	−13	(−23 to 49)	0.488	−34	(−13 to 82)	0.178
MI^3)^	−16	±	21	−12	±	22	−3	(−56 to 64)	0.899	−6	(−48 to 58)	0.808	−11	(−41 to 63)	0.694	−24	(−34 to 80)	0.441
**Energy intake-dinner (kcal)**																		
LOCF^1)^	−23	±	6	−4	±	6	−19	(−35 to −3)	0.031	−19	(−35 to −2)	0.040	−19	(−35 to −3)	0.030	−29	(−48 to −10)	0.007
CDS^2)^	−29	±	7	2	±	8	−31	(−51 to −11)	0.008	−31	(−51 to −11)	0.007	−37	(−58 to −16)	0.003	−41	(−61 to −20)	0.001
MI^3)^	−30	±	10	−7	±	11	−23	(−51 to 5)	0.136	−22	(−50 to 6)	0.147	−24	(−50 to 3)	0.110	−24	(−54 to 4)	0.114
**Proportion of carbohydrate to total energy intake (%)**																		
LOCF^1)^	0.0	±	0.3	0.6	±	0.4	0.6	(−0.4 to 1.6)	0.246	0.7	(−0.3 to 1.6)	0.193	0.6	(−0.3 to 1.5)	0.205	0.2	(−1.0 to 1.3)	0.768
CDS^2)^	0.0	±	0.4	−0.5	±	0.4	0.4	(−0.6 to 1.5)	0.373	0.4	(−0.5 to 1.3)	0.390	0.3	(−0.5 to 1.2)	0.438	−0.3	(−1.1 to 0.6)	0.559
MI^3)^	−0.1	±	0.7	0.0	±	0.7	0.2	(−2.0 to 2.0)	0.883	0.0	(−2.0 to 2.1)	0.985	−0.1	(−1.8 to 2.0)	0.945	−0.3	(−1.7 to 2.3)	0.764
**Proportion of protein to total energy intake (%)**																		
LOCF^1)^	0.2	±	0.1	0.2	±	0.1	0.0	(−0.3 to 0.3)	0.837	0.0	(−0.4 to 0.3)	0.764	0.1	(−0.4 to 0.2)	0.759	0.1	(−0.3 to 0.5)	0.633
CDS^2)^	0.3	±	0.1	0.3	±	0.1	0.0	(−0.4 to 0.4)	0.999	0.0	(−0.4 to 0.3)	0.862	0.0	(−0.4 to 0.4)	0.884	0.2	(−0.1 to 0.6)	0.257
MI^3)^	0.3	±	0.2	0.1	±	0.2	−0.2	(−0.9 to 0.5)	0.512	−0.2	(−0.8 to 0.5)	0.546	−0.2	(−0.8 to 0.5)	0.597	−0.3	(−0.3 to 0.6)	0.825
**Proportion of total fat to total energy intake (%)**																		
LOCF^1)^	−0.2	±	0.3	0.3	±	0.3	−0.6	(−1.3 to 0.1)	0.152	−0.6	(−1.3 to 0.1)	0.111	−0.6	(−1.2 to 0.1)	0.110	−0.3	(−1.2 to 0.5)	0.435
CDS^2)^	−0.2	±	0.3	0.2	±	0.3	−0.5	(−1.3 to 0.4)	0.282	−0.4	(−1.1 to 0.3)	0.287	−0.3	(−0.9 to 0.3)	0.307	0.1	(−0.6 to −0.7)	0.861
MI^3)^	−0.2	±	0.6	−0.2	±	0.6	0.0	(−1.9 to 1.8)	0.755	−0.1	(−1.5 to 1.6)	0.941	0.0	(−1.4 to 1.5)	0.953	0.1	(−1.6 to 1.4)	0.900
**Vegetable intake-whole day (g)**																		
LOCF^1)^	35.1	±	5.5	−0.2	±	5.7	35.3	(19.6 to 50.6)	0.000	29.0	(14.9 to 43.1)	0.001	28.6	(14.3 to 42.9)	0.001	25.5	(5.9 to 45.1)	0.021
CDS^2)^	43.2	±	6.3	5.4	±	7,1	37.8	(19.2 to 56.4)	0.001	31.4	(13.2 to 49.6)	0.003	30.1	(11.6 to 48.7)	0.005	28.6	(8.9 to 48.2)	0.011
MI^3)^	39.1	±	8.5	−1.4	±	9.3	40.5	(13.2 to 67.9)	0.007	31.6	(8.5 to 56.7)	0.021	31.6	(7.8 to 55.6)	0.024	30.5	(5.6 to 55.4)	0.034
**Vegetable intake-breakfast (g)**																		
LOCF^1)^	16.0	±	2.8	−0.3	±	2.8	16.3	(8.6 to 23.9)	0.001	13.0	(5.9 to 20.0)	0.002	12.7	(6.5 to 18.9)	0.001	14.3	(4.6 to 23.7)	0.001
CDS^2)^	20.2	±	3.0	0.8	±	3.3	19.4	(10.5 to 28.3)	0.000	14.1	(6.3 to 21.9)	0.003	13.1	(5.3 to 20.9)	0.004	11.5	(1.1 to 21.5)	0.043
MI^3)^	15.5	±	4.2	−0.5	±	4.5	16.0	(3.9 to 28.3)	0.021	13.0	(−0.3 to 22.5)	0.078	12.7	(−0.2 to 21.3)	0.079	12.5	(−0.3 to 22.7)	0.062
**Vegetable intake-lunch (g)**																		
LOCF^1)^	13.1	±	3.0	0.5	±	3.1	9.5	(4.2 to 21.0)	0.009	9.9	(2.2 to 16.9)	0.020	9.0	(1.7 to 16.4)	0.027	6.0	(4.3 to 16.2)	0.270
CDS^2)^	15.9	±	3.4	5.1	±	3.8	10.8	(10.8 to 20.8)	0.048	8.5	(−0.4 to 17.5)	0.078	8.0	(−1.2 to 17.2)	0.108	3.6	(−8.0 to 15.1)	0.550
MI^3)^	15.0	±	4.5	−0.3	±	4.9	15.3	(0.6 to 30.0)	0.042	10.9	(−2.3 to 24.1)	0.113	10.5	(−1.3 to 22.2)	0.106	9.8	(−2.1 to 21.7)	0.134
**Vegetable intake-dinner (g)**																		
LOCF^1)^	6.4	±	2.8	−0.6	±	2.9	7.2	(−0.8 to 14.8)	0.095	5.8	(−1.5 to 13.1)	0.129	5.6	(−1.7 to 12.9)	0.144	4.2	(−4.8 to 13.3)	0.376
CDS^2)^	5.7	±	3.1	0.1	±	3.4	5.6	(−0.8 to 16.9)	0.093	6.0	(−2.4 to 14.9)	0.177	6.2	(−14.3 to 45.1)	0.174	3.1	(−5.9 to 45.1)	0.491
MI^3)^	8.3	±	3.9	−1.3	±	4.3	9.7	(−3.4 to 19.8)	0.132	8.2	(−3.4 to 19.8)	0.186	8.0	(−4.6 to 20.7)	0.196	8.2	(−3.8 to 20.2)	0.205
**Dietary fiber intake (g)**																		
LOCF^1)^	0.7	±	0.2	0.1	±	0.2	0.6	(0.1 to 1.0)	0.022	0.5	(0.1 to 0.9)	0.039	0.5	(−0.1 to 1.0)	0.051	0.4	(0.0 to 1.0)	0.241
CDS^2)^	0.9	±	0.2	0.3	±	0.2	0.6	(0.0 to 1.2)	0.087	0.4	(−0.1 to 1.0)	0.157	0.4	(−0.2 to 1.0)	0.225	0.3	(−0.5 to 1.0)	0.489
MI^3)^	0.7	±	0.3	0.1	±	0.3	0.6	(−1.6 to 0.4)	0.246	0.4	(−1.4 to 0.5)	0.346	0.4	(−1.4 to 0.6)	0.380	0.4	(−1.3 to 0.6)	0.393

SE: standard error, 95%CI: 95% confidence interval, degree of freedom = 18.

^#^ Value of HbA_1C_ is JDS^15^.

1) LOCF: last observation carried forward (IG: n = 100, CG: n = 93).

2) CDS: complete data set. (IG: n = 84, CG: n = 70).

3) MI: Multiple imputation with all analyzed variables (number of imputations = 200) (IG: n = 100, CG: n = 93).

Model 1: crude.

Model 2: mixed model adjusted for baseline.

Model 3: mixed model adjusted for baseline, gender, age and BMI.

Model 4: mixed model adjusted for baseline, gender, age, BMI, smoking status, exercise status, change of exercise level, family history of type 2 diabetes, and complication.

## Discussion

This 6-month cluster randomized controlled trial to assess the effectiveness of a structured individualized lifestyle education program aimed at modifying dietary intake by meal for type 2 diabetes patients in primary care clinics successfully registered 193 participants. The ITT/LOCF analysis to compare the mean changes at 6 months from baseline in the HbA_1c_ level showed a significantly greater reduction in the IG compared to the CG (−0.5%, p = 0.004). The sensitivity analyses by baseline adjusted analyses of PPA/CDS and ITT/MI showed similarly significant effectiveness. In the analyses of dietary intake, compared with the CG, in the IG energy intake at dinner decreased significantly from baseline and vegetable intake significantly increased for the whole day, breakfast, and lunch in the all models. These results were confirmed by several sensitivity analyses, whereas statistical significance was not shown for the other clinical and dietary outcome measures. These results suggested that the structured individual-based lifestyle education by registered dietitians for glycemic control in primary care settings has the potential to improve HbA_1c_ levels in patients with type 2 diabetes.

To encourage reduction in energy intake at dinner and increase in vegetable intake at breakfast and lunch is useful in lowering the HbA_1c_ level to improve glycemic control with type 2 diabetes patients not only in Japan but also in other countries. Furthermore, although not significant, the proportion of patients achieving the recommended clinical target for HbA_1c_ (<6.9%) [10] was greater in the IG (45%) than in the CG (29%).

Although mean changes between the two groups were not significant for BMI, this might be an understandable result considering that ‘normal’ BMI range for Asians is less than other groups of Europeans and Americans [19,20], and thus are at risk of developing type 2 diabetes patients at a lower BMI. Asking patients who have already reduced total energy intake to some extent, even though not to a sufficient extent, to make further reductions may be not feasible and usually results in low compliance. This is particularly so in patients who are aware of their limitations for weight control or who are not obese and therefore have a moderate energy intake. To change the way of eating at dinner and to recommend eating more vegetables may bring about greater compliance for participants whether or not they are obese. In turn, our dietary education by modifying dietary intake according to meals and encouraging vegetable intake has the probability of being effective in Asian patients who are not obese. In Japan, lifestyle education by registered dietitians for diabetes patients is provided mainly in hospitals and in only a few clinics. Dietary lifestyle education is important for many of type 2 diabetes patients. To conduct a system for effective dietary lifestyle education in clinics should be considered to be warranted in Japan.

### Comparison with other studies

The clinical effectiveness of several medical nutrition therapies for diabetes has been reported [21,22]. Research is increasingly demonstrating that medical nutrition therapy administered by a registered dietitians or nutrition professional is a key component of diabetes management and complements treatment of diabetes by physicians [22]. Considering results from past studies [5,6,16,23]–[32], it is important for effective glycemic control 1) that patients be given results of nutritional assessments so that meals can be modified [5,6,16], 2) that a registered dietitian provides support to patients in establishing reasonable goals [5,6,16,23,26,28], and 3) that patients be given continuous support in behavior modification [5,6,23-32]. Because medical doctors usually do not have the time for nutritional education, support by co-medical staff members such as registered dietitians is important to bring successful nutritional education to fruition. Actually, several randomized controlled trials examining the effects of long-term lifestyle education by co-medical staff, including dietary education, resulted in success in decreasing HbA_1c_ levels [6,23,26,27,31]. Studies [23,27,31] with baseline HbA_1c_ levels (7.3% to 7.7%) similar to ours (7.5%) showed an effect size for the mean change from baseline of −0.7%, -0.5%, and −0.1%, respectively, which did not differ largely from our result of −0.5%.

Because excessive eating at night increases insulin resistance [35], the SILE program aimed to improve FPG by reducing energy intake at dinner and to improve post-meal plasma glucose by increasing vegetable intake at breakfast and lunch and increasing dietary fiber intake. It is known that targeting both postprandial plasma glucose and FPG is an important strategy for achieving optimal glycemic control [36]. The SILE program is characterized as having a target that can be continued by the participant and that would improve both FPG and postprandial plasma glucose levels.

### Strengths and limitations of the study

Studies on lifestyle interventions in individuals with type 2 diabetes have been performed in recent years, including those with individual randomization [5,6,23,24,26-29,31] and cluster randomization designs [25,30,32-34,37,38]. It must be considered that in clinic-based studies there is the possibility of contamination bias between intervention and control participants in the same clinic [39]. Thus, a cluster randomization design would eliminate this possibility. To our knowledge, this is the first cluster randomized study to evaluate structured individual-based lifestyle education in a community setting over a 6-month treatment period in Japan.

To modify behavior with regard to dietary intake, an individualized approach is required in addition to information on appropriate dietary content. In this study, we could review in detail a patient’s dietary habits using the FFQW82 and graphs, which helped patients to recognize their nutritional problems and decide what problem-solving and goal-setting strategies could be implemented. This may have successfully resulted in reduced energy intake at dinner and increased vegetable intake at breakfast and lunch and an improvement in the HbA_1c_ level in the IG.

As to whether plasma glucose was more often monitored in the IG than in the CG, there is little possibility of increased monitoring in the IG. In Japan, almost all patients who use insulin are likely to use home glucose monitoring, however, the proportions of patients using insulin were similar (around 10%) in each group, which could eliminate a significant bias.

There are some limitations in this study design. Firstly, only patients were blinded to the assignment of education. In order to avoid selection bias, we asked general practitioners to recruit all the patients in turn. Furthermore, the low number of registered patients in the CG was mainly because of an extended sick leave of a general practitioner in charge of one clinic and the inclusion of a newly started clinic (enrollment of 10 patients, respectively). Therefore, the potential selection bias may be slight.

Secondly, the dropout rates were relatively small; 20% for the IG and 25% for the CG. Because our study was based on usual clinical situations, patients were expected to visit their clinics every 2 or 3 months, and we did not anticipate a large number of dropouts. Even though the protocol required enrollment of 10 patients per clinic, we permitted enrollment of 7–13 patients per clinic to allow for dropouts so that statistical power could be maintained and to take into account a shortfall in enrollment in some clinics. Furthermore, we conducted multiple imputation analyses to examine the effect of dropouts on the results.

General practitioners were randomly assigned to either the IG or CG, and general practitioners were permitted to follow their usual clinical practices. Changes in medicines by the participants were not numerous and proportions of these changes were not largely different between the IG and CG (n = 7; 7.0% and n = 11; 11.8%, respectively. See Figure 1). We cannot deny the possibility that the general practitioners of patients with less than optimal glycemic control might have changed the diabetes medication of some of these patients during the study period, which would bring about a bias. However, changes in diabetes medication are inevitable in the management of patients with less than optimal glycemic control. In order to examine this point, we added a sensitivity analysis that included 18 patients whose medications were changed during the study period. Thus, PPA included 172 patients (91 [91%] IG, 81 [87%] CG). The results were almost concordant with results shown for patients who did not have changes in medication during the study period. In addition, none of the patients used dietary fiber pills as a supplement; however, 4 patients took a tea that was considered a health food to maintain postprandial glucose (2 for IG and 2 for CG). This would not seem to affect vegetable intake.

Thirdly, the success of this program was to some degree dependent on the skills of the dietitians involved. To address this issue, we developed a training process for the registered dietitians before the start of the randomization study. Education in implementing the program was therefore important. Furthermore, the assessment sheet with items ranked according to priority helped standardize advice by the registered dietitians. Training for the registered dietitians was conducted based on an instruction manual and thus did not differ between dietitians at the intervention clinics and the control clinics. Although the effects were somewhat varied among the dietitians, the variability among those assigned to the IG and CG was similar. Furthermore, we mentioned that five registered dietitians were in charge of both groups. However, because the dietitians were trained to give advice following the manual, the possibility of contamination of advice was less likely. As for advice on activity, the IG had received more practical advice compared to the CG. In fact, 37% of the IG responded that they had performed more exercise compared to the CG (17%). Therefore, not only dietary education but also practical advice on activity might have affected the results. However, our results showed an improvement in dietary intakes, and it is natural to make the interpretation that improvement in dietary intakes resulted in improvements in plasma glucose levels.

Fourthly, in order to improve glycemic control, maintaining long-term control is required. An earlier assessment could be biased as a result of changes made only because subjects were conscious of being studied. The follow-up period in this study was 6 months. However, further study is warranted.

### Implication

In the past, simple lifestyle education for patients with type 2 diabetes was conducted by a general practitioner or clinical nurse in community medical settings while providing medical care. Although dietary education by registered dietitians has been increased by degrees, continuous evidence-based lifestyle education should be warranted. Active utilization of dietitians as co-medical staff can help in successfully providing diabetes care in community medicine. This study provides some evidence for the value of structured individual-based lifestyle education for glycemic control by registered dietitians in primary care settings. The result will be useful to encourage lowering HbA_1c_ and it may help to improve glycemic control not only in Japan but also in other countries.

## Conclusions

The dietitians delivered structured individual-based lifestyle education for glycemic control in primary care settings resulted in significantly improved HbA_1c_ levels in the participants with type 2 diabetes. These results will provide useful information to not only Japanese type 2 diabetes patients but also to other Asian type 2 diabetes patients as well as non-obese type 2 diabetes patients in Western countries.

## Abbreviations

BMI: Body mass index; CDS: Complete data set; CG: Control group; FPG: Fasting plasma glucose; FFQ: Food frequency questionnaire; FFQW82: Food frequency questionnaire consisting of a list of 82 foods; HbA1c: Hemoglobin A_1c_; IG: Intervention group; ITT: Intention-to-treat; JDS: Japan Diabetes Society; LOCF: Last observation carried forward; MAR: Missing at random; MI: Multiple imputation; NGSP: National Glycohemoglobin Standardization Program; PPA: Per protocol analysis; RCT: Randomized controlled trial; SILE: Structured individual-based lifestyle education

## Competing interests

The authors declare that they have no competing interests.

## Authors’ contributions

MA, KY, MW, MN, EH, and TT conceived and designed the study. IK provided support for medical issue. MA and MW supervised lifestyle education. MA and KY had full access to all of the data in the study and take responsibility for the integrity of the data and the accuracy of the data analysis. MA, KY, and TT analyzed and interpreted the data and drafted the manuscript. All authors have seen and approved the final version of the paper for publication. KY and TT provided administrative support and supervised the study.

## Pre-publication history

The pre-publication history for this paper can be accessed here:

http://www.biomedcentral.com/1471-2458/13/467/prepub
